# Improving Compliance With the Sepsis Six Care Bundle in the Surgical Emergency Department: A Closed-Loop Clinical Audit

**DOI:** 10.7759/cureus.115139

**Published:** 2026-08-25

**Authors:** Basit Ali, Qirat H Aizdee, Umair Ahmad Munir, Muhammad Atif Kanjoo, Faizan E Mustafa

**Affiliations:** 1 General Surgery, Nishtar Medical University, Multan, PAK; 2 Thoracic Surgery, Nishtar Medical College and Hospital, Multan, PAK; 3 Surgery, Nishtar Medical College and Hospital, Multan, PAK; 4 Pulmonology, Shaikh Zayed Medical College and Hospital, Multan, PAK; 5 Internal Medicine, Nishtar Medical University, Multan, PAK

**Keywords:** closed-loop audit, emergency department, quality improvement, sepsis bundle, sepsis six

## Abstract

Background: Sepsis is one of the most important causes of morbidity and mortality and is a disease that affects more patients in low- and middle-income countries. Sepsis Six interventions are six time-critical interventions that can be delivered within an hour of sepsis recognition and have been shown to decrease mortality when reliably implemented, but adherence has been poor globally, especially in resource-challenged environments.

Objective: The aim of this study was to evaluate and enhance adherence to the Sepsis Six care bundle in adult patients presenting with sepsis in a tertiary care hospital in Pakistan through a closed-loop clinical audit approach.

Methods: The study method adopted was a prospective closed-loop clinical audit in Nishtar Hospital in Multan. Sixty consecutive adult patients with sepsis were evaluated for baseline compliance with the six elements of the Sepsis Six package in Cycle 1 (October 2, 2024, to January 3, 2025). After identification of an area of need, a structured intervention phase was put in place, which included educational sessions for medical and nursing staff, sepsis-awareness teaching, and the introduction of a standard sepsis proforma. A further 60 consecutive patients underwent re-audit of compliance, using the same criteria, in Cycle 2 (April 4, 2025, to July 5, 2025). Compliance rates, timing for individual interventions, and documentation quality were compared between cycles using the chi-square test (or Fisher's exact test where expected cell counts were <5) and the Mann-Whitney U test for non-normally distributed timing data.

Results: There was no difference in baseline characteristics between the two audit cycles. There was a significant improvement after the intervention in all of the above, which stayed significant after Holm-Bonferroni correction for multiple comparisons (p ≤ 0.005). The key outcome of delivering the Sepsis Six bundle within one hour significantly increased from 15/60 (25.0%) to 42/60 (70.0%) (absolute difference 45.0%, 95% CI 27.5%-58.7%; χ² = 24.36, df = 1; OR 7.00, 95% CI 3.13-15.64; φ = 0.45). Median time to first antibiotic administration was reduced from 95 to 50 minutes (Mann-Whitney U ≈ 1174, Z ≈ −3.29, p < 0.001, r = 0.30), with documentation of sepsis increasing from 18/60 (30.0%) to 51/60 (85.0%) (p < 0.0001).

Conclusion: A low-cost educational package, combined with a structured sepsis proforma, was associated with significant and clinically meaningful improvements in compliance with the Sepsis Six bundle in a resource-limited tertiary hospital. The results confirm the value and feasibility of the closed-loop audit as a quality improvement tool applicable to other environments and the need for continued training and regular re-audits to sustain the benefits of the closed-loop audit.

## Introduction

Sepsis is considered a life-threatening organ dysfunction resulting from a dysregulated host response to infection [1]. According to the consensus definition of Sepsis-3, this is defined as evidence of infection plus an acute rise of ≥2 points on the Sequential Organ Failure Assessment (SOFA) score [1]. Formal SOFA scoring will, however, require laboratory/blood gas parameters that may not be available at first contact in resource-limited emergency settings, and therefore bedside screening tools are commonly used to identify at-risk patients for expedient assessment and pathway activation, as described in more detail in the Methods section. It claimed approximately 11 million lives in 2017, accounting for approximately 20% of all deaths worldwide [2], and it was responsible for the bulk of these deaths in low- and middle-income countries. Sepsis is associated with high morbidity and mortality in South Asian countries in comparison to high-income countries due to late presentation, lack of critical care infrastructure, and non-adherence to evidence-based sepsis care bundles [3,4]. The Sepsis Six care bundle is an initiative of the UK Sepsis Trust and is derived from the Surviving Sepsis Campaign resuscitation bundle [5,6]. It comprises three diagnostic and three therapeutic interventions. These include high-flow oxygen, taking blood cultures (considering source control), providing intravenous broad-spectrum antibiotics, providing intravenous fluid resuscitation, measuring blood lactate levels, and monitoring urine output every hour [5,6]. All interventions are to be completed within one hour of recognition of sepsis [5,7]. Reliable delivery of the bundle has been associated with a reduction in physiological derangement [7], hospital stay, and death due to sepsis [5].

The Sepsis Six bundle has been in use for more than a decade, but difficulties with implementation remain in high-income and resource-poor areas. Audits in the United Kingdom report overall compliance with the full bundle in emergency departments and general wards to be low, ranging from approximately 4% to 14% in reported studies, even in institutions with long-term implementation [8,9]. A multicentre UK surgical audit revealed low uptake of the full bundle for patients with severe sepsis (4.8%), with the most commonly missing elements being oxygen delivery and blood culture collection [10]. In Portugal, only 22% of patients with severe sepsis or septic shock received the complete resuscitation bundle, and high levels of compliance were associated with increased survival [11]. In a Korean hospital-based cohort study, earlier completion of individual bundle components was associated with lower hospital mortality, indicating that timing within the care cycle may be as important as bundle completion [12]. Evidence from Pakistan remains limited. Poor compliance with individual bundle elements was observed in a retrospective audit at a tertiary teaching hospital in Karachi, where less than half of the patients had serum lactate levels measured [13]. A recent Pakistani quality improvement initiative also showed a marked enhancement in the implementation of Sepsis Six following a structured educational intervention as well as a decrease in mortality, intensive care unit (ICU) admission rates, and hospital length of stay [14].

To our knowledge, no study from Pakistan has adopted the “closed-loop” approach to clinical audit (pre-specified clinical audit standard, structured re-audit, formal statistical comparison of the audit results at each audit cycle), which is the most rigorous approach to clinical audit. Closed-loop audit has been seen as the most successful form of clinical audit as it can provide evidence of the impact of corrective action taken as a result of a clinical audit, rather than just a picture of the audit at a given time [15]. As such, a two-cycle closed-loop clinical audit was carried out at Nishtar Hospital, Multan, with the aim of finding out the percentage of patients receiving complete delivery of the Sepsis Six bundle within one hour from the time of recognition of sepsis, before and after a structured education and documentation-based intervention. Secondary objectives were to determine compliance with individual bundle components and timeliness of key interventions. This audit was not aimed at the clinical efficacy or patient-centered outcomes of the care process but rather at compliance with the Sepsis Six standard.

## Materials and methods

Study design and setting

This was a prospective, two-cycle, closed-loop audit of the Emergency Department and surgical emergency admission units of Nishtar Hospital, a large tertiary care teaching hospital in a vast catchment population in South Punjab, Pakistan, and affiliated with Nishtar Medical University. The audit was conducted in a standard clinical audit cycle, which involved data collection, comparison with a set standard, implementation of corrective intervention, and re-audit of the set standard [15].

Sample size

This was a clinical audit for quality improvement to assess the implementation of the Sepsis Six bundle in usual clinical practice, not to test a predetermined hypothesis of efficacy. So a formal sample size calculation was not done. Rather, each eligible adult patient who presented to the Emergency Department or surgical admission unit was recruited during the audit periods until 60 patients were recruited in each cycle. The approach adopted agrees with the general guidelines of clinical audit practice (pragmatic sampling of consecutive cases is recommended to get an unbiased picture of the current practice and to enable comparison after corrective interventions have been carried out).

Standard

The criteria used were the active provision of all six elements of the Sepsis Six care bundle within one hour of sepsis being recognised: oxygen administered to correct hypoxaemia (targeting SpO₂ 94-98%, or 88-92% in patients at risk of hypercapnic respiratory failure), blood cultures before giving any antibiotics, broad-spectrum intravenous antibiotics, intravenous fluid resuscitation, serum lactate level, and monitoring urine output hourly, as recommended by the UK Sepsis Trust and Surviving Sepsis Campaign [5,6,16]. Patients who were normoxic at baseline (and thus supplemental oxygen was not clinically indicated) were noted as compliant without the actual administration of supplemental oxygen. “Time zero” was defined as the time Systemic Inflammatory Response Syndrome (SIRS)/qSOFA thresholds were first documented as met (Cycle 2: on the sepsis proforma), or, where not explicitly time-stamped, the time of the earliest qualifying vital sign or laboratory value; all 60 patients per cycle had a determinable time zero and were included in the denominator for all bundle-compliance and timing outcomes.

Population

Eligible patients were adults (≥18 years) with clinical suspicion or confirmation of infection, together with evidence of a dysregulated host response, defined as meeting either of the following: (a) two or more SIRS criteria [17], comprising a temperature greater than 38°C or less than 36°C, a heart rate greater than 90 beats per minute, a respiratory rate greater than 20 breaths per minute or PaCO₂ <32 mmHg (4.3 kPa), or a white blood cell count >12,000/mm³ or <4,000/mm³ or >10% immature bands; and/or (b) a quick SOFA (qSOFA) score of 2 or more [1,18], based on a respiratory rate of 22 breaths per minute or more, altered mental status (Glasgow Coma Scale score <15), and a systolic blood pressure of 100 mmHg or less. These tools were used as pragmatic screening criteria to trigger the institutional sepsis pathway and enrollment in this audit, rather than as formal Sepsis-3 diagnostic criteria, which require organ dysfunction to be confirmed using the SOFA score. This approach was adopted because full SOFA parameters (arterial blood gases, bilirubin, creatinine, and platelet count) were not routinely or rapidly available at initial presentation in our setting and because the audit aimed to assess bundle compliance in patients managed as septic in routine clinical practice rather than to derive a strict Sepsis-3 cohort. There were 60 consecutive eligible patients in each audit cycle. Patients were excluded if they had already had a sepsis bundle initiated at another facility, had incomplete clinical records, died, or left the hospital before the sepsis bundle could be evaluated.

The SIRS criteria and qSOFA score are published in the peer-reviewed literature and are available for clinical and research use freely, with no requirement to obtain a license or permission [17,18]. A structured sepsis proforma used for data collection, clinical documentation, and staff education (described below) was developed in-house specifically for this audit, has not been published elsewhere, and is repeated in full in the Appendices.

Cycle 1 (baseline audit)

Cycle 1 ran from October 2, 2024, to January 3, 2025. A structured proforma was used to gather data on the time of sepsis recognition (time zero) and the time to delivery of each of the six bundle elements from clinical notes, observation charts, laboratory results, and pharmacy records.

Intervention Phase

After baseline audit analysis, the findings were presented to the Department of Surgery and Emergency Department teams in a departmental audit meeting. Key strengths were the early identification of sepsis, adequate adherence to the majority of the Sepsis Six bundle, and early initiation of intravenous antibiotic therapy. Key weaknesses identified included the delayed recognition of sepsis, suboptimal compliance with some aspects of the Sepsis Six bundle, delayed application of intravenous antibiotics, poor monitoring of urine output, and failure to complete sepsis documentation. A varied quality improvement intervention was conducted in the following months. This comprised educational sessions, visual reminders, standardized sepsis proforma, and audit feedback.

Educational sessions: Interactive teaching sessions with junior doctors, house officers, medical officers, postgraduate trainees, and nurses in the Emergency Department and surgical admission units. The sessions included the diagnostic criteria for recognizing sepsis (SIRS and qSOFA), the significance of the “golden hour,” and the correct sequence and timely completion of all six elements of the Sepsis Six bundle.

Visual reminders: Early recognition and early management of the Sepsis Six were reinforced by displaying laminated Sepsis Six algorithms at strategic places in the Emergency Department, resuscitation area, and surgical admission units as reminders.

Standardized sepsis proforma: A proforma was introduced to the hospital to document the time that sepsis was recognized (“time zero”), the time taken to complete each Sepsis Six step, and continuous monitoring of the patient. The proforma was designed to be a clinical documentation tool and also as a checklist to avoid elements of the clinical bundle from being omitted.

Audit feedback: Results of the baseline audit were reported to medical and nursing personnel at departmental meetings. Poor compliance areas were discussed, barriers to implementing timely interventions were identified, and practical approaches were agreed to improve adherence to the Sepsis Six bundle.

No extra staff, equipment, or funds were added during the intervention period. The above interventions for quality improvement (educational sessions, visual reminders, the standardized sepsis proforma, and audit feedback) were implemented as one structured rollout over the four weeks following the Cycle 1 audit meeting (mid-January to early February 2025) and were then maintained without further changes in the structure for the next ~10 weeks leading up to and during Cycle 2 data collection (to July 2025). After the initial rollout, there were no further teaching sessions or reinforcement activities to test the sustainability of one low-intensity algorithm package rather than multiple doses.

Cycle 2 (re-audit)

Cycle 2 was conducted from April 4 to July 5, 2025, using the same inclusion criteria, data collection proforma, and sampling method (60 consecutive eligible patients), and was thus directly comparable.

Outcome Measures

The primary outcome was the percentage of patients receiving each component of the Sepsis Six bundle within one hour of sepsis recognition (“time zero”). The study also examined how many patients received all six parts of the bundle within that first hour.

Secondary outcomes were the median time to administration of intravenous antibiotics, intravenous fluid resuscitation, serum lactate measurement, blood culture collection, and completeness of the sepsis-specific documentation, as well as the use of the standardized sepsis proforma after the implementation of the quality-improvement intervention.

As this project was designed to be a clinical audit to assess adherence to evidence-based care processes, patient-centered clinical outcomes (mortality, intensive care unit admission, length of hospital stay, readmission) were not prespecified or collected.

Statistical analysis

This project is not a hypothesis-driven study; however, the closed-loop audit report provides inferential statistics to describe the magnitude and precision of observed changes. Data were saved in Microsoft Excel (Microsoft Corporation, Redmond, Washington) and then analyzed with IBM SPSS Statistics for Windows, Version 31 (Released 2025; IBM Corp., Armonk, New York). Categorical variables were presented as frequencies and percentages and compared between audit cycles using the Pearson chi-square test. Fisher's exact test was used when expected cell counts were fewer than five. The p-value is followed by the chi-square statistic (df), the odds ratio (OR) (95% CI), and the phi (φ) coefficient (standardized effect size measure for 2 × 2 tables). The Newcombe hybrid score (Wilson) method was used to determine 95% confidence intervals for differences in proportions between audit cycles. Multiple comparisons were made for the six different bundle components and the combined outcome, and p-values were corrected for multiple comparisons using the Holm-Bonferroni method; results were reported as significant only when the p-value survived the correction. Means ± SD (standard deviation) were used to report continuous variables when normally distributed. Time-to-intervention variables were not normally distributed and were therefore reported as medians; comparisons between audit cycles were made using the Mann-Whitney U test, with the U statistic, Z value, and effect size (r) reported for each comparison in Table 3. The Shapiro-Wilk test was used to check normality. The absolute percentage-point improvements were calculated for each of the Sepsis Six bundle components. All tests were two-tailed, and p < 0.05 was used to determine statistical significance. If any data were missing for the primary audit results, the results for that outcome were not used in the analysis. No imputation of missing data was done.

Effect sizes were calculated as follows. For 2 × 2 categorical comparisons, the phi coefficient was calculated as follows:

$\varphi = \sqrt{\frac{\chi^{2}}{N}}$

where χ² is the Pearson chi-square statistic, and N is the total sample size for that comparison. For Mann-Whitney U comparisons, the effect size r was calculated as follows:

$r = \frac{Z}{\sqrt{N}}$

where Z is the standardized test statistic from the Mann-Whitney U test, and N is the total number of observations across both compared groups.

## Results

Baseline characteristics

Demographic and clinical data of patients in the two audit cycles are summarized in Table 1. Age, sex distribution, comorbidity profile, source of infection, and severity of presentation (qSOFA ≥ 2 and serum lactate > 2 mmol/L) were similar between the two cohorts, making a direct before-and-after comparison appropriate.

**Table 1:** Baseline characteristics of patients included in the clinical audit

Characteristic	Audit Cycle 1 (n = 60)	Audit Cycle 2 (n = 60)
Age (years), mean ± SD	52.8 ± 16.2	54.1 ± 15.7
Male sex, n (%)	37 (61.7%)	39 (65.0%)
Female sex, n (%)	23 (38.3%)	21 (35.0%)
Comorbidities, n (%)		
Diabetes mellitus	21 (35.0%)	19 (31.7%)
Hypertension	18 (30.0%)	20 (33.3%)
Chronic kidney disease	5 (8.3%)	6 (10.0%)
Malignancy	6 (10.0%)	5 (8.3%)
Immunosuppression	4 (6.7%)	5 (8.3%)
Source of infection, n (%)		
Intra-abdominal infection	25 (41.7%)	24 (40.0%)
Soft tissue infection	10 (16.7%)	11 (18.3%)
Biliary sepsis	9 (15.0%)	10 (16.7%)
Perforated viscus	6 (10.0%)	5 (8.3%)
Urological source	5 (8.3%)	6 (10.0%)
Other sources	5 (8.3%)	4 (6.7%)
Severity at presentation		
qSOFA ≥2, n (%)	32 (53.3%)	34 (56.7%)
Serum lactate >2 mmol/L, n (%)	27 (45.0%)	29 (48.3%)

Compliance with the individual Sepsis Six elements

The percentage of compliance with each of the Sepsis Six bundle components was significantly higher in Cycle 2 than in Cycle 1 (Table 2) for all seven measures, with absolute improvements ranging from 21.7% (IV fluid resuscitation) to 45.0% (complete bundle delivery within one hour). IV fluid resuscitation increased from 42/60 (70.0%) to 55/60 (91.7%) (χ² = 9.09, df = 1; OR 4.71, 95% CI 1.62-13.73), IV antibiotic use within 1 hour of sepsis recognition (time zero) increased from 38/60 (63.3%) to 54/60 (90.0%) (χ² = 11.93, df = 1; OR 5.21, 95% CI 1.93-14.07), and blood cultures were collected before antibiotic use in an increasing proportion, from 32/60 (53.3%) to 49/60 (81.7%) (χ² = 10.98, df = 1; OR 3.90, 95% CI 1.70-8.92). Oxygen administration increased from 28/60 (46.7%) to 48/60 (80.0%) (χ² = 14.35, df = 1; OR 4.57, 95% CI 2.03-10.29). The proportion of patients with a serum lactate level measured increased from 30/60 (50.0%) to 50/60 (83.3%) (χ² = 15.00, df = 1; OR 5.00, 95% CI 2.14-11.66), and the proportion with urine output monitored increased from 24/60 (40.0%) to 46/60 (76.7%) (χ² = 16.59, df = 1; OR 4.93, 95% CI 2.24-10.86). Overall, complete Sepsis Six bundle delivery within one hour increased from 15/60 (25.0%) in Cycle 1 to 42/60 (70.0%) in Cycle 2, representing an absolute improvement of 45.0% (95% CI 27.5% to 58.7%; χ² = 24.36, df = 1; OR 7.00, 95% CI 3.13-15.64). Effect sizes across the seven comparisons ranged from φ = 0.28 to φ = 0.45, corresponding to medium-to-large effects. Full effect sizes, confidence intervals, and unadjusted and multiplicity-adjusted p-values for each comparison are listed in Table 2.

**Table 2:** Compliance with individual Sepsis Six components before and after intervention χ² = Pearson chi-square statistic; OR = odds ratio (Cycle 2 vs Cycle 1); φ = phi coefficient (effect size for 2×2 tables). p-values shown are from Fisher's exact test; Holm-adjusted p is the Holm-Bonferroni corrected p-value across the seven comparisons.

Sepsis Six Component	Audit Cycle 1 (n=60)	Audit Cycle 2 (n=60)	Absolute Improv. (95% CI)	χ² (df=1)	OR (95% CI)	φ	Fisher's exact p	Holm-adj. p	Still signif.?
Oxygen administered	28/60 (46.7%)	48/60 (80.0%)	+33.3% (16.2% to 47.9%)	14.35	4.57 (2.03–10.29)	0.346	0.00027	0.00109	Yes
Blood cultures before antibiotics	32/60 (53.3%)	49/60 (81.7%)	+28.4% (11.6% to 43.0%)	10.98	3.90 (1.70–8.92)	0.302	0.00164	0.00327	Yes
IV antibiotics within 1 hour of recognition	38/60 (63.3%)	54/60 (90.0%)	+26.7% (11.7% to 40.4%)	11.93	5.21 (1.93–14.07)	0.315	0.00097	0.00291	Yes
IV fluid resuscitation	42/60 (70.0%)	55/60 (91.7%)	+21.7% (7.6% to 35.0%)	9.09	4.71 (1.62–13.73)	0.275	0.00462	0.00462	Yes
Serum lactate measured	30/60 (50.0%)	50/60 (83.3%)	+33.3% (16.6% to 47.6%)	15.00	5.00 (2.14–11.66)	0.354	0.00019	0.00095	Yes
Urine output monitored	24/60 (40.0%)	46/60 (76.7%)	+36.7% (19.2% to 51.1%)	16.59	4.93 (2.24–10.86)	0.372	0.00008	0.00050	Yes
Complete Sepsis Six within 1 hour	15/60 (25.0%)	42/60 (70.0%)	+45.0% (27.5% to 58.7%)	24.36	7.00 (3.13–15.64)	0.451	<0.0001	0.00005	Yes

After applying the conservative Holm-Bonferroni correction for multiple testing, all seven comparisons were statistically significant (adjusted p < 0.005 for all comparisons), suggesting that the improvements were great and reliable, even after multiple testing corrections.

Timing and documentation

Table 3 shows that the timeliness of delivery of key interventions was significantly shorter on each measure in Cycle 2. The median time to first antibiotic administration was reduced from 95 minutes in Cycle 1 to 50 minutes in Cycle 2 (Mann-Whitney U = 1174, Z = -3.29, p < 0.001, r = 0.30). The median time to IV fluid resuscitation was reduced from 55 minutes to 30 minutes (U = 1212, Z = -3.09, p = 0.002, r = 0.28). The median time to serum lactate measurement was reduced from 80 minutes to 35 minutes (U = 1174, Z = -3.29, p < 0.001, r = 0.30). The median time to blood culture collection was reduced from 45 minutes to 25 minutes (U = 1252, Z = -2.88, p = 0.004, r = 0.26). Sepsis-specific documentation was completed in only 18/60 (30.0%) of Cycle 1 patients, rising to 51/60 (85.0%) in Cycle 2 (+55.0%, p < 0.0001), coinciding with introduction of the standardized sepsis proforma, which was used in 0/60 (0%) of Cycle 1 patients (as it did not yet exist) and 45/60 (75.0%) of Cycle 2 patients (+75.0%, p < 0.0001).

**Table 3:** Timing of key interventions and documentation quality Mann-Whitney U test for continuous timing variables; Pearson chi-square/Fisher's exact test for documentation and proforma use.

Measure	Cycle 1	Cycle 2	Difference	Mann-Whitney U	Z	Effect size r	p-value
Median time to first antibiotic	95 minutes	50 minutes	−45 min	1174	−3.29	0.30	<0.001
Median time to IV fluids	55 minutes	30 minutes	−25 min	1212	−3.09	0.28	0.002
Median time to lactate measurement	80 minutes	35 minutes	−45 min	1174	−3.29	0.30	<0.001
Median time to blood culture	45 min	25 min	−20 min	1252	−2.88	0.26	0.004
Sepsis documentation completed, n (%)	18/60 (30.0%)	51/60 (85.0%)	+55.0%	_	_	_	<0.0001
Sepsis proforma used, n (%)	0/60 (0%)	45/60 (75.0%)	+75.0%	_	_	_	<0.0001

## Discussion

This targeted clinical audit showed that a multifaceted quality improvement intervention was associated with a significant improvement in compliance with the Sepsis Six bundle in surgical and emergency admissions at a tertiary care hospital in Pakistan in a closed-loop fashion. The primary outcome, timely delivery of the Sepsis Six bundle (within one hour), improved from 15/60 (25.0%) to 42/60 (70.0%) (absolute improvement 45.0% (95% CI, 27.5%-58.7%), χ² = 24.36, df = 1, p < 0.0001). There were also large improvements in each of the individual bundle elements (oxygen administration, blood culture collection, intravenous antibiotics, intravenous fluid resuscitation, serum lactate measurement, and urine output monitoring). Staff education, visual reminders, standardized documentation, and regular audit feedback also significantly reduced median time to antibiotic administration and other time-sensitive interventions. These results show that there is potential for significant improvement in adherence to evidence-based sepsis care with relatively simple and low-cost interventions in resource-limited health systems.

Our results were similar to the Pakistani study by Baig et al., which showed poor adherence to sepsis resuscitation bundle components in a tertiary care emergency department, especially in terms of prompt blood culture and lactate level measurement [13]. The same was true of our audit in the baseline phase, with compliance in some bundle elements being somewhat higher. Our study used a closed-loop audit design, allowing targeted corrective action, followed by reassessment of clinical practice, unlike the cross-sectional assessment reported by Baig et al. This marked improvement in the second audit cycle shows that most deficiencies in early sepsis management are due to factors beyond clinical knowledge, which are modifiable through systems. A parallel quality improvement program undertaken contemporaneously by Shafquat et al. showed a similar improvement in Sepsis Six compliance, from 29.3% to 73.0%, after a similar educational and protocol-based approach, and also noted a reduction in hospital length of stay, intensive care unit admission (32.0% to 21.0%), and mortality (27.0% to 17.0%) [14]. The consistency of our results with those of Shafquat et al. further reinforces the confidence that such low-cost, structured quality improvement interventions can reliably improve Sepsis Six compliance in various tertiary care hospitals in Pakistan. There are, however, some significant differences: Shafquat et al. were not formal about their closed-loop audit design, which lacked a predefined re-audit standard; our study also uses robust confidence intervals and multiplicity-adjusted significance testing. Both of these studies were unable to make any definitive statement about causality between improved compliance and improved patient-centered outcomes; however, because no concurrent control groups were used in either study, the reduction in mortality seen by Shafquat et al. is remarkable but needs to be confirmed by well-powered, multicenter studies before it can be concluded that there is any evidence of survival benefit.

Our findings also align with previous quality improvement efforts reported by Burke et al., who showed that the Sepsis Six bundle, with the use of specific sepsis equipment and Critical Care Outreach services, resulted in a significant improvement in the completion of the sepsis bundle [7]. Our institution did not have any other specialist teams or dedicated sepsis equipment. Still, great strides were made in sepsis management through education, standardizing sepsis documentation, reminder posters, and audit feedback. The results indicate that these organized interventions offer the potential for sustainable improvements in sepsis care at little or no financial cost, which is critical for healthcare systems in low- and middle-income countries. Although sepsis guidelines have been widely disseminated, studies in other countries have consistently reported poor compliance with the Sepsis Six bundle. Frankling et al., Baldwin et al., the UK National Surgical Collaborative, and Carvas et al. reported low levels of complete implementation of the bundles, especially regarding the prompt administration of oxygen, the collection of blood cultures, prompt measurement of serum lactate, and the delivery of antibiotics [8,10,11,19]. While there are differences in healthcare infrastructure, patient populations, and audit methodology between the two, the results of our baseline study were similar to these reports. It is important to note, however, that compliance rates in our study were comparable to those of several published audit studies, indicating that structured quality-improvement strategies are effective in enhancing adherence to recommended sepsis care pathways. Similarly, Hyun et al. found that use of a standardized electronic sepsis response protocol was associated with earlier completion of individual bundle components, notably blood culture, which was associated with lower hospital mortality rates [12]. Our results corroborate these conclusions, as we found that pragmatic interventions that are specific to local clinical practice can be used in a tertiary care hospital to deliver improvements in Pakistan. The strong consistency between findings across healthcare systems indicates that education, standardization, and ongoing audit and feedback are universal strategies for enhancing early sepsis management.

There are many clinical implications of the present study. First, it shows that clinical audit can significantly improve adherence to international sepsis guidelines when undertaken routinely, accompanied by timely feedback and appropriate education and training. Second, the intervention was not associated with any extra staffing, special equipment, or investment of large sums of money; therefore, it is easily replicable in other resource-poor hospitals. Lastly, this is one of the first closed-loop clinical audits to assess the implementation of the Sepsis Six bundle published in Pakistan, thus bringing locally relevant evidence to support continuous quality-improvement programs to improve sepsis care. Future multicenter studies that measure patient-centered outcomes, for example, mortality, intensive care unit admission, and hospital length of stay, should be undertaken to establish whether the process measures are translated into better clinical outcomes.

Limitations

This audit has some limitations. First, it was carried out at one center with a relatively small number of patients (60 per cycle), which may not be generalizable to other centers in Pakistan. Second, the time difference between completion of Cycle 1 and commencement of Cycle 2 introduces the possibility of confounding by the Hawthorne effect, as a general awareness of the auditing process could have increased the reported quality of care, that is, the documented practice, unrelated to the actual audit. Staff turnover and seasonal variation in case mix may also have introduced confounding. Third, importantly, this audit report shows process compliance improvements only and does not show that this led to improvements in patient-centered outcomes like survival, which should not be assumed from our data. Fourth, as in most bundle-compliance audits, we cannot completely rule out documentation bias: the more care is documented (on the new proforma), the greater the increase in compliance that is measured. Fifth, the sustainability of the improvement beyond the Cycle 2 audit window was not considered, and further audits will need to be undertaken to ensure that the gains are sustained. Lastly, time zero was reconstructed retrospectively from clinical notes in Cycle 1 but recorded prospectively on the proforma in Cycle 2, which may itself have contributed to the apparent improvement in timeliness. Additionally, sepsis was operationally identified using SIRS/qSOFA screening criteria rather than formal Sepsis-3 SOFA-based diagnosis; this pragmatic approach reflects real-world resource constraints but may have included some patients who would not meet strict Sepsis-3 criteria or missed others with organ dysfunction not captured by qSOFA.

## Conclusions

This closed-loop clinical audit shows that an easy-to-implement, low-cost intervention package of staff education, visual prompts, and a standardized sepsis proforma was associated with large, statistically significant improvements in compliance with the Sepsis Six care bundle in a resource-limited tertiary hospital in Pakistan, with the proportion of complete one-hour Sepsis Six bundle delivery increasing from 15/60 (25.0%) to 42/60 (70.0%). Because of the uncontrolled before-and-after design, these results are considered evidence of local process-of-care improvement. They should not be considered to demonstrate clinical effectiveness or generalizability to other low- and middle-income settings, which should not be assumed without further study. Embedding sustainable systems (ongoing training, checklist-based documentation, and repeated re-audit) can support the sustainability and further development of these benefits. Efforts ought to be made to evaluate the longevity of this improvement and, through adequately powered comparative studies, its causal effect on mortality and other clinical outcomes in future research.

## Figures and Tables

**Figure 1:**
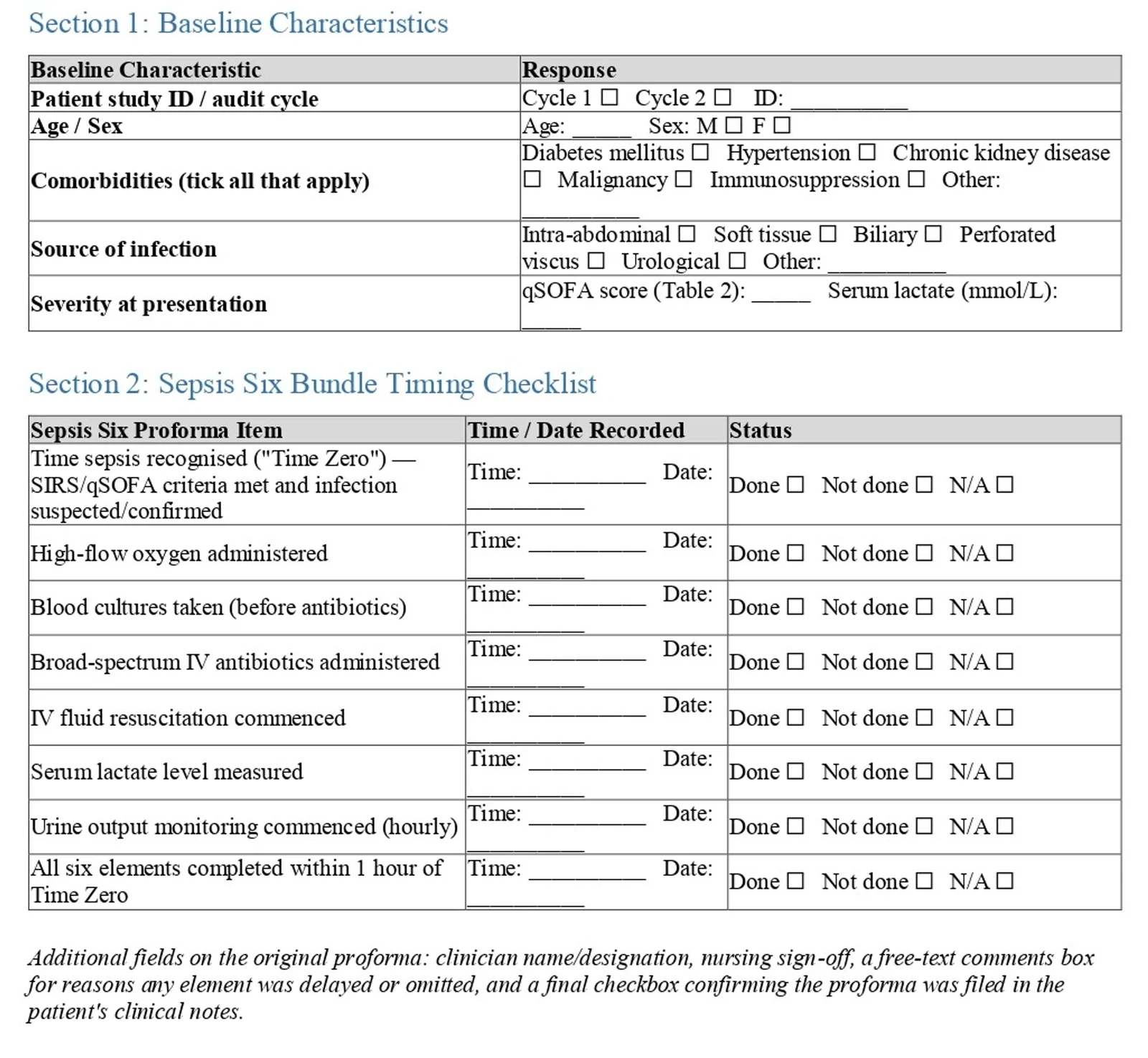
Structured sepsis proforma

## Appendices

The following proforma was developed in-house by the study authors and introduced during the intervention phase (prior to Cycle 2) as a clinical documentation tool and checklist for the Sepsis Six bundle.

## References

[1] Singer M, Deutschman CS, Seymour CW (2016). The Third International Consensus definitions for sepsis and septic shock (Sepsis-3). JAMA.

[2] Rudd KE, Johnson SC, Agesa KM (2020). Global, regional, and national sepsis incidence and mortality, 1990-2017: analysis for the Global Burden of Disease Study. Lancet.

[3] Raza HA, Hashmi AP, Khakwani MM, Ali MH, Jamil B (2024). Review of sepsis in Pakistan: how far have we come?. IJID Reg.

[4] Stephen AH, Montoya RL, Aluisio AR (2020). Sepsis and septic shock in low- and middle-income countries. Surg Infect (Larchmt).

[5] Daniels R, Nutbeam T, McNamara G, Galvin C (2011). The sepsis six and the severe sepsis resuscitation bundle: a prospective observational cohort study. Emerg Med J.

[6] (2025). UK Sepsis Trust. Healthcare professionals. https://sepsistrust.org/healthcare-professionals/.

[7] Burke J, Wood S, Hermon A, Szakmany T (2019). Improving outcome of sepsis on the ward: introducing the 'Sepsis Six' bundle. Nurs Crit Care.

[8] Frankling C, Patel J, Sharif B, Melody T, Yeung J, Gao F, Szakmany T (2019). A snapshot of compliance with the sepsis six care bundle in two acute hospitals in the West Midlands, UK. Indian J Crit Care Med.

[9] Kopczynska M, Unwin H, Pugh RJ (2021). Four consecutive yearly point-prevalence studies in Wales indicate lack of improvement in sepsis care on the wards. Sci Rep.

[10] UK National Surgical Research Collaborative (2017). Multicentre observational study of adherence to Sepsis Six guidelines in emergency general surgery. Br J Surg.

[11] Carvas JM, Canelas C, Montanha G, Silva C, Esteves F (2016). Impact of compliance with a sepsis resuscitation bundle in a Portuguese emergency department. Acta Med Port.

[12] Hyun DG, Lee S, Choi S, Son J, Park SH, Hong SB, Lim CM (2025). Impact of the National Early Warning Score-based sepsis response system on hospital-onset sepsis in a tertiary hospital in South Korea. Acute Crit Care.

[13] Baig MA, Sheikh S, Hussain E, Waheed S, Mian A (2018). Compliance with the sepsis resuscitation care bundles in the emergency department of a developing world tertiary care hospital - a clinical audit. Infect Dis J Pak.

[14] Shafquat Y, Ujjan ID, Shankar R, Kumar S, Rai L, Soothar K (2026). Implementation and compliance with the Sepsis Six bundle in the emergency department: a quality improvement project in a resource-limited tertiary care setting. Pak J Pathol.

[15] Hart A (2002). Principles for best practice in clinical audit. Postgrad Med J.

[16] Evans L, Rhodes A, Alhazzani W (2021). Surviving sepsis campaign: international guidelines for management of sepsis and septic shock 2021. Intensive Care Med.

[17] Bone RC, Balk RA, Cerra FB (1992). Definitions for sepsis and organ failure and guidelines for the use of innovative therapies in sepsis. The ACCP/SCCM Consensus Conference Committee. American College of Chest Physicians/Society of Critical Care Medicine. Chest.

[18] Seymour CW, Liu VX, Iwashyna TJ (2016). Assessment of clinical criteria for sepsis: for the Third International Consensus Definitions for sepsis and septic shock (Sepsis-3). JAMA.

[19] Baldwin LN, Smith SA, Fender V, Gisby S, Fraser J (2008). An audit of compliance with the sepsis resuscitation care bundle in patients admitted to A&E with severe sepsis or septic shock. Int Emerg Nurs.

